# Dual-Sensitive Gold-Nanocubes Platform with Synergistic Immunotherapy for Inducing Immune Cycle Using NIR-Mediated PTT/NO/IDO

**DOI:** 10.3390/ph15020138

**Published:** 2022-01-25

**Authors:** Hsin-Yi Tsao, Hung-Wei Cheng, Chia-Chi Kuo, San-Yuan Chen

**Affiliations:** 1 Materials Science and Engineering, National Yang Ming Chiao Tung University, Hsinchu 300, Taiwan; yvonne880217@gmail.com (H.-Y.T.); mlb14756@gmail.com (H.-W.C.); d42172000@gmail.com (C.-C.K.); 2 Graduate Institute of Biomedical Science, China Medical University, Taichung 404, Taiwan; 3 Frontier Research Centre on Fundamental and Applied Sciences of Matters, National Tsing Hua University, Hsinchu 300, Taiwan; 4 School of Dentistry, College of Dental Medicine, Kaohsiung Medical University, Kaohsiung 807378, Taiwan

**Keywords:** S-nitrosoglutathione (GSNO), gold nanocubes (AuNCs), photothermal therapy (PTT), IDO immunotherapy

## Abstract

Currently, the combination therapies based on immunotherapy have been rapidly developed, but the response rate has not always increased as expected. Nano-platform has become a potential strategy which can trigger multi-functions to increase immunotherapeutic efficacy via activating T-cells and photothermal effect. Herein, to avoid the self-degradation and provide pH-sensitive property, S-nitrosoglutathione (GSNO) was loaded in gold nanocubes (AuNCs) with polyacrylic acid (PAA) coating. Subsequently, the layer-by-layer (LbL) assembly of iron oxide nanoparticles (Fe_3_O_4_) and betanin can provide the conjugation of 1-methyl-D-tryptophan (1-M-DT) on the nanoparticle to form an NO gas-photothermal-immune nano-platform (GAPFBD) for achieving combinatory therapy of NO gas, photothermal therapy (PTT), and indoleamine 2,3-dioxygenase (IDO) immunotherapy. After irradiation by 808-nm laser, the GSNO was released under a lower pH environment due to the structural transformation of PAA and then transformed into NO production of 64.5 ± 1.6% under PTT. The combination of PTT and NO gas therapy can effectively eliminate cancer cells, resulting in a large amount of tumor-associated antigens (TAAs) compared to the individual treatment in vitro. Additionally, the released 1-M-DT inhibited IDO and combined with TAAs to enhance maturation of dendritic cells (DCs), indicating the excellent synergistic effect of PTT and NO with IDO inhibitors. These results revealed that this dual-sensitive nanoparticle presented a combination strategy of PTT/NO/IDO for the synergistic effect to promote DC maturation.

## 1. Introduction

With the advancement and development of nanomedicine, different innovative technologies such as gene therapy, photothermal therapy (PTT), photodynamic therapy (PDT), and immunotherapy have been gradually applied to various cancer treatments. In recent years, combination therapies based on the use of various types of immune modulators have been developed, including indoleamine 2,3-dioxygenase (IDO) immunotherapy [1], CAR-T [2], immune checkpoint [3], and. Among them, IDO inhibitors can inhibit the degradation of tryptophan (Trp) and activate T cells to produce an immune response through regulating the balance of Trp and kynurenine (Kyn) [1,4]. However, IDO inhibitors are not able to induce effective in vivo IDO inhibition due to their low in vitro potency and the similar plasma concentrations of the IDO substrate [5]. To solve this problem, recent studies have demonstrated that the use of the nano-platform to deliver the IDO inhibitors can significantly enhance the therapeutic effect. For instance, Chen et al. used a nanoplatform to deliver the IDO inhibitor NLG919 and paclitaxel, indicating that the combination treatment presented better tumor suppression than the single treatment group [6]. In addition, Xing et al. also proved that this combination using photodynamic therapy (PDT) and IDO inhibitor can produce an apparent effect on long-term immune memory [7]. The above-mentioned studies indicated that the use of nano-formulated IDO inhibitors combined with other therapies has great potential for achieving efficacious therapies. However, PDT is usually limited by the strong demand for oxygen, resulting in ineffectively treating the hypoxic area of the tumor.

Currently, nitric oxide (NO), a common biological signal molecule in organisms, has several tumoricidal effects, such as inhibiting DNA synthesis, activating caspase protein, and promoting cell apoptosis [8,9,10]. The destruction of cancer cells by NO is gradually regarded as an alternative to the current research on PDT to treat cancer. At the same time, NO contains free radicals, which are a member of highly reactive nitrogen species (RNS) and can regulate a variety of cancer-related reactions in tumor tissues [11]. In addition, NO is also an important bioactive molecule exerting multiple functions in many physiological and pathological processes, including the ability to functionally inhibit the activity of IDO [8,12]. NO has been proven to promote cell apoptosis to reach the anti-tumor effects at high concentrations [9,10]. More importantly, NO can enhance the sensitivity of chemotherapy and irradiation therapy and further reconstruct the blood vessel to improve the recruitment of immune cells [11]. Nonetheless, the extremely short half-life of NO seriously limits its therapeutic effect [13], and high reactive NO can cause cell death and tissue damage for normal cells due to exposure to high concentrations of NO [14]. Recently, NO donors have been developed for decreasing the toxicity of NO in the use of NO gas therapy. For example, S-nitrosoglutathione (GSNO) [15] is one of the NO donors which has been used in various nano-platforms to increase the effective accumulation in targeted tissues and tumors [16]. Additionally, GSNO is an endogenous species that can be decomposed into NO gases and glutathione disulphide (GSSG) by heat and UV/visible light. After the reaction is triggered, the generated NO can react with reactive oxygen species (ROS) to produce RNS, which boosts the destructive activity and enhances the overall damage [17,18]. Therefore, various thermal-response nano-platforms have been designed for loading GSNO to release NO gases at an accurate time. For example, Zhang et al. developed the upconversion nanoparticles (UCNPs) combined with photosensitive NO donors to modify its structure and release NO [19]. However, the UCNPs with low drug-loading and low energy-conversion efficiency greatly limits the application and development of this system.

**Scheme 1:**
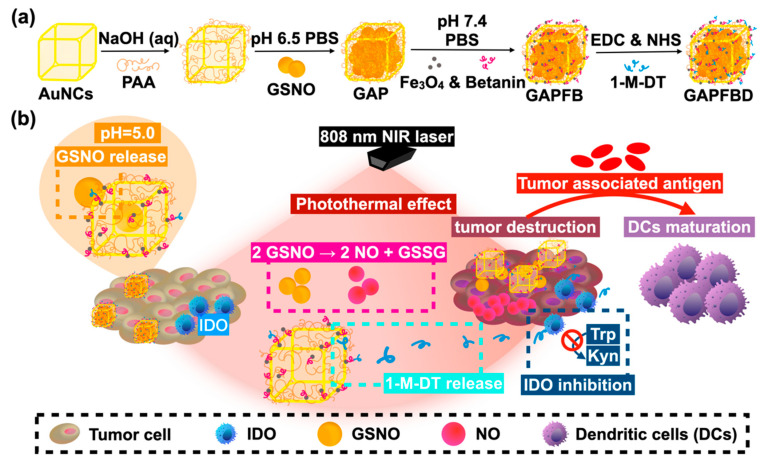
Schematic illustration of (**a**) the synthesis of GSNO-AuNCs-Fe_3_O_4_-betanin-1-M-DT (GAPFBD) nanoparticle and (**b**) the synergistic mechanisms of GAPFBD in tumor treatment.

Gold nanocubes (AuNCs) are a photothermal conversion nano-material [20,21] that have been widely used as the agent of PTT to induce the ablation of the targeted tissue. AuNCs can also be a promising nano-platform for GSNO because they can absorb specific irradiation wavelengths for generating heat to induce the production of NO gas. In addition, NO gas can fundamentally overcome the challenge of the production of heat shock proteins under thermal stimulation after PTT [22]. Hence, in this study, we developed a dual-sensitive nano-platform using AuNCs to load GSNO and combine with inhibitory properties of IDO to increase the synergistic effect for achieving the combination therapy. During the synthesis of the dual-sensitive nanoparticle, GSNO-loaded AuNCs were first encapsulated within polyacrylic acid (PAA), followed by coating iron oxide nanoparticles (Fe_3_O_4_), betanin, and 1-methyl-D-tryptophan (1-M-DT) through layer-by-layer assembly to build up a NO gas-photothermal-immune nano-platform in Scheme 1a. GSNO was triggered by the PTT for the production of NO gas. PAA is a pH-sensitive polymer coated on the surface of AuNCs through the anion of PAA and the cation of AuNCs [5,23]. It can not only protect GSNO from leaking but also control the accurate release location. Fe_3_O_4_ as magnetic navigation can deliver the nanoparticle to the tumor site. Betanin is a water-soluble red beet extract that can be degraded under high temperatures [24] to promote the release of 1-M-DT. The treatment mechanism of the NO gas-photothermal-immune nanoplatform was illustrated in Scheme 1b. First, GSNO can be released from the GAPFBD at the intracellular level due to the structural transformation of PAA at pH = 5.0. Then, the dual-sensitive nanoparticle can simultaneously initiate multiple treat mechanisms triggered by 808-nm laser irradiation to achieve the synergistic effects. Under 808-nm laser irradiation, the photothermal effect was induced by GAPFBD to generate heat for accelerating the dissolution of GSNO into NO gas. The heat can also trigger the release of 1-M-DT to suppress the degradation of Trp into Kyn. Finally, tumor-associated antigens (TAAs) can be induced to enhance the maturation of dendritic cells (DCs), causing the activation of T cells. This demonstrates that the GAPFBD has great potential multi-functions to promote the maturation of dendritic cells (DCs) for achieving long-term immunity to enhance tumor treatment. 

## 2. Results and Discussion

### 2.1. Characterization of GSNO-AuNCs-PAA-Fe_3_O_4_-Betanin-1-M-DT (GAPFBD) 

**Figure 1:**
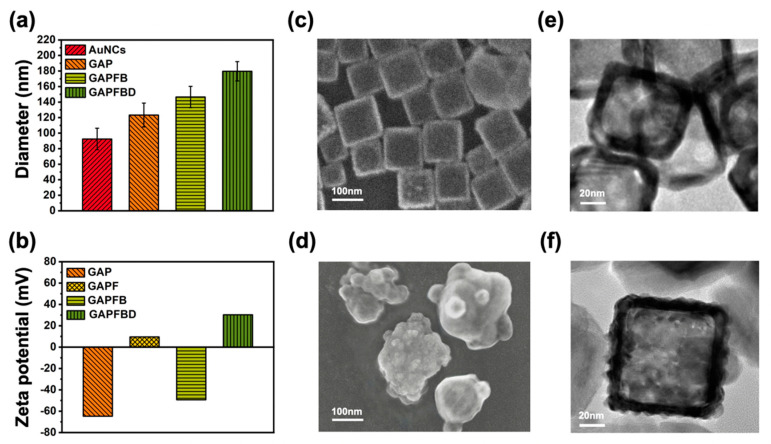
Characteristics of GAPFBD. (**a**) DLS of AuNCs, GAP, GAPFB and GAPFBD. (**b**) Zeta potential of GAP, GAPF, GAPFB, and GAPFBD. SEM image of (**c**) AuNCs and (**d**) GAPFBD. TEM image of (**e**) AuNCs and (**f**) GAPFBD.

The synthesis and characterization of the GSNO-AuNCs-PAA-Fe_3_O_4_-betanin-1-M-DT (GAPFBD) were first analyzed. The GSNO was loaded in AuNCs by controlling the pH, and subsequently, GSNO-loaded AuNCs were encapsulated within polyacrylic acid (PAA) to form GSNO-AuNCs-PAA(GAP) through the anion of PAA with the metal cation under alkaline conditions. Next, the iron oxide (Fe_3_O_4_) and betanin were coated on the surface of GAP sequentially through the layer-by-layer technique to form composite GSNO-AuNCs-PAA-Fe_3_O_4_-betanin (GAPFB). Finally, the 1-methyl-D-tryptophan (1-M-DT) was grafted to the GAPFB via EDC/NHS reaction to form the GAPFBD. During the synthesis of GAPFBD, the particle size, zeta potential, and morphology of AuNC-based nanoparticles were further analyzed. As shown in Figure 1a, the particle sizes measured by dynamic light scattering (DLS) increased gradually from 92.5 ± 13.9 nm of AuNCs, 123.3 ± 15.4 nm of GAP, 146.5 ± 3.7 nm of GAPFB to 179.7 ± 23.4 nm of GAPFBD. Further, to ensure that the nanoparticle can be fabricated via layer-by-layer processing, the zeta potential of GAP, GAPF, GAPFB, and GAPFBD was measured as −64.73 mV, +9.55 mV, −49.33 mV, and +30.31 mV in Figure 1b, respectively. The results confirmed that the GSNO-AuNCs-PAA-Fe_3_O_4_-betanin-1-M-DT (GAPFBD) nanoparticle can be successfully synthesized using AuNCs through the layer-by-layer assembly. Moreover, the morphology and dispersion of the AuNCs can be observed by SEM and TEM as shown in Figure 1c,e, respectively, illustrating that AuNCs presented the square shapes. The SEM and TEM of GAPFBD in Figure 1d,f indicated the AuNCs were coated with multiple-layers to form a complex structure. The Fe_3_O_4_ coating on the GAP can be confirmed using TEM imaging and energy-dispersive X-ray spectroscopy (EDS), as shown in Figure S1. The atomic percentage of Fe and Au was measured as 5.48% and 85.38% in Table S1. Furthermore, the encapsulation efficiency of GSNO was 74.42 ± 4.14% and the conjugating ratio of 1-M-DT was 74.00 ± 1.41% based on the standard curve in Figure S2, respectively. The stability of GAPFBD was also conducted by placing the sample in pH = 7.4 and pH = 6.0 PBS storage conditions in Figure S3.

### 2.2. Thermally Dependent NO Production and pH-Responsive Drug Release

**Figure 2:**
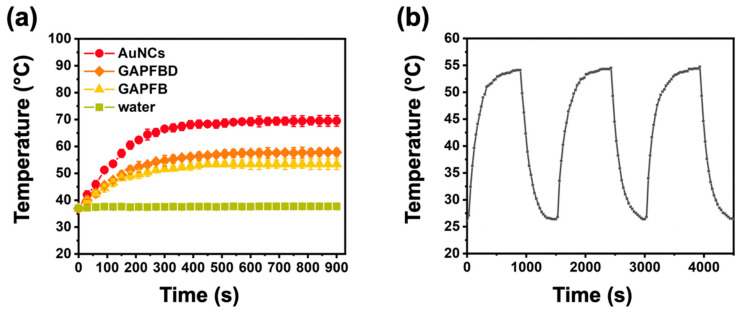
Photothermal effect of AuNC-based nanoparticles. (**a**) Heating curve of various nanoparticles under NIR starting from 37 °C and (**b**) Photothermal cycling stability of GAPFBD over three on/off cycles of NIR laser irradiation.

The photothermal effect of GAPFB depends on the absorption of incident photons of AuNCs that convert photon energy into heat through optical excitation [25]. To evaluate the photothermal effect of GAPFB, the temperature curve of water, AuNCs, GAPFB, and GAPFBD was measured under NIR laser irradiation (808 nm, 0.5 W/cm^2^) for 15 min in Figure 2a. The starting temperature was set at 37 °C to imitate the body temperature. Without AuNCs, the temperature of water changed slightly from 37 °C to 38 °C. In contrast, AuNCs presented an apparent change in the temperature from 37 °C to 69 °C in 10 min, while the temperature change in GAPFB was measured from 37 °C to 53 °C, lower than AuNCs. The maximum temperature difference of AuNCs and GAPFBD was attributed to the multi-layer coating on AuNCs to reduce the thermal effect of GAPFBD. Here, it was noted that the GAPFBD can reach 45 °C within 2 min, indicating that GAPFBD still presented a better photothermal effect [26,27]. In addition, the temperature elevation after three cycles of NIR irradiation did not decrease in Figure 2b, indicating that GAPFBD possessed excellent photothermic stability, which was beneficial to perform repeated photothermal therapy in long-term clinical treatments. 

**Figure 3:**
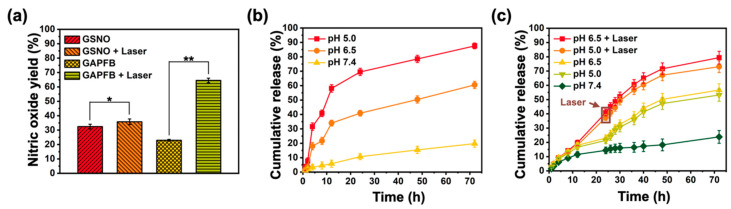
(**a**) NO production with/without NIR laser. (**b**) GSNO release under different pH conditions. (**c**) 1-M-DT release under different pH conditions with/without NIR laser. Data are expressed as mean ± SD, *n* = 3. Two-tailed Student’s test was used. * *p* < 0.05, ** *p* < 0.01.

To prove that GSNO-AuNCs-PAA-Fe_3_O_4_-betanin (GAPFB) can control GSNO to produce NO gas, an NIR laser was applied to raise the temperature [28]. Figure 3a shows the NO production of GSNO and GAPFB with/without NIR laser irradiation (0.5 W/cm^2^, 10 min) as measured by Nitric Oxide Assay Kit and calculated by the standard curve in Figure S4. GSNO produced 32.4 ± 1.6% of NO due to the self-degradation of GSNO. As GSNO was irradiated with NIR laser, 35.8 ± 1.97% NO was generated, indicating NIR laser displayed little effect on the generation of NO. In contrast, GAPFB produced a lower NO of 22.9 ± 0.6% than GSNO, which was attributed to the fact that GSNO was well encapsulated in the GAPFB. However, with NIR laser treatment, the GAPFB produced a much higher NO of 64.5 ± 1.6%. The results confirmed that the conversion from GSNO into NO gas can be well triggered by GAPFB under NIR irradiation. In addition, to confirm that GAPFB can control the release of GSNO through PAA, the GSNO release was performed at various times of 1, 2, 4, 8, 12, 24, 48, and 72 h under different pH values, including systemic circulation (pH = 7.4), tumor microenvironment (pH = 6.5), and intracellular (pH = 5) in Figure 3b. GSNO released 19.8 ± 3.0% at pH = 7.4 in 72 h, indicating that GSNO was effectively encapsulated. In contrast, GSNO was compatibly released as 60.5 ± 2.5% at pH = 6.5 and 87.6 ± 2.0% at intracellular pH = 5.0, respectively, because the structure of PAA was opened at a lower pH value. The results demonstrated the increased release of GSNO under a lower pH condition. Moreover, in order to confirm that GAPFBD can release 1-M-DT in intracellular and tumor microenvironments, GAPFBD was separately placed at pH = 7.4, 6.5, and 5.0, with/without NIR laser treatments in Figure 3c. The released amount of 1-M-DT was measured at about 23.8 ± 4.5% at pH = 7.4, 56.7 ± 4.4% at pH = 6.5, and 53.2 ± 4.3% at pH = 5.0 in 72 h. In addition, as NIR laser irradiation was applied at the time point of 24 h for 10 min at pH = 5.0 and pH = 6.5, it can be observed that the released amount of 1-M-DT was apparently increased by about 15% and finally reached 73.0 ± 4.1% and 79.4 ± 4.5% after 72 h, respectively. The results demonstrated that the release of 1-M-DT from the GAPFBD can be controlled by NIR laser irradiation under different pH environments.

### 2.3. Cellular Uptake

**Figure 4:**
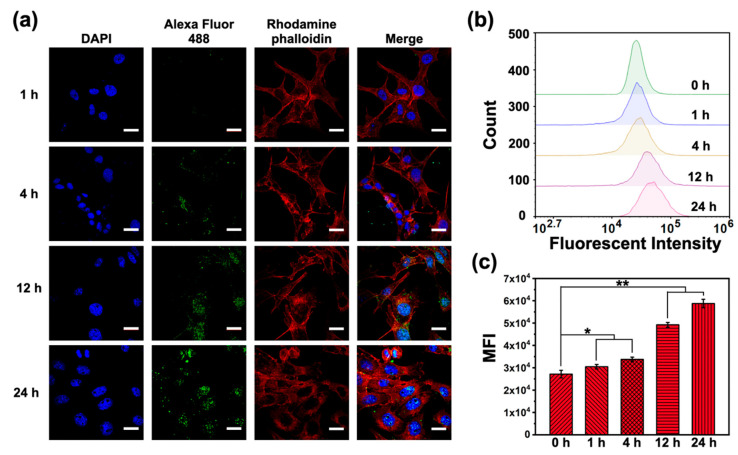
Cellular uptake of GAPFB. (**a**) Fluorescence microscopy image. DAPI (blue) and rhodamine phalloidin (red) were used for staining the nuclei and cytoskeleton, respectively. Green indicated GAPFB. (Scale bar = 100 μm) (**b**) Flow cytometric analysis and (**c**) quantified results. Data are expressed as mean ± SD, *n* = 3. Two-tailed Student’s test was used. * *p* < 0.05, ** *p* < 0.01.

To ensure the optimal time point of the maximum GSNO release under the NIR treatment, the intracellular uptake of GAPFB was observed at various incubation times. The nuclei and cytoskeleton of the Hep55.1c were stained by DAPI (blue) and rhodamine phalloidin (red), respectively. As shown in Figure 4a, GAPFB, as indicated in green, appeared around the cell at 4 h compared to 1 h. Moreover, the intensity of green fluorescence presented an obvious increase within nuclei in 12 h and 24 h. The endocytosis behavior can be further confirmed using flow cytometry in Figure 4b,c. The mean fluorescent intensity (MFI) of the cell relative to GAPFB was observed after 1 h and increased with the incubation time. The maximum cellular uptake amount of the GAPFB appeared at 24 h so that it was selected for subsequent cell experiments.

### 2.4. In Vitro NO/Photothermal Treatment and Apoptosis Analysis

**Figure 5:**
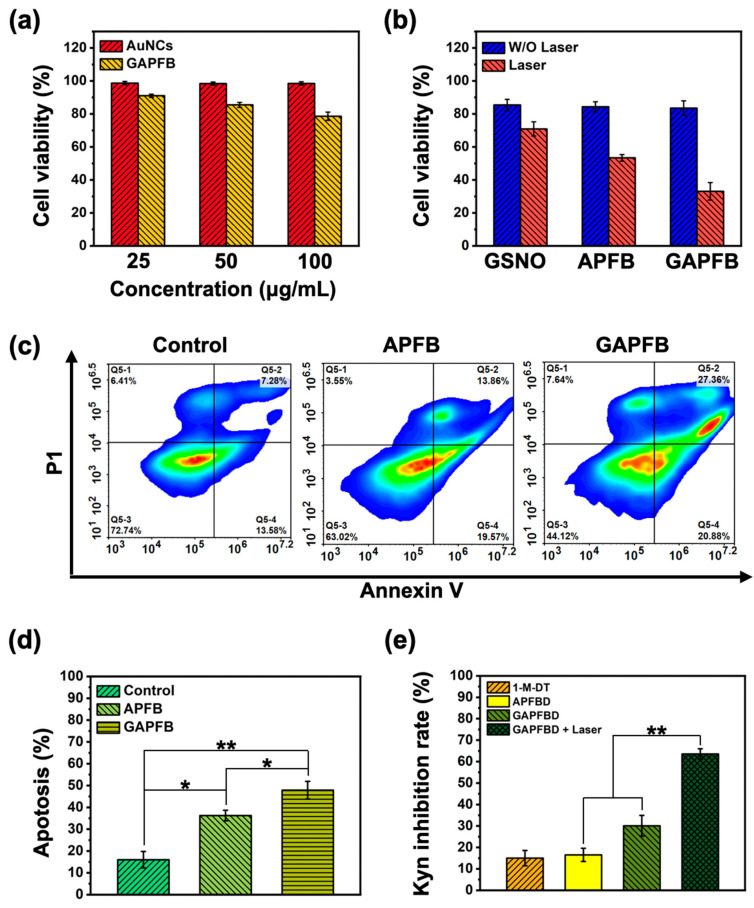
(**a**) Cytotoxicity assay for AuNCs and GAPFB in Hep55.1c without NIR laser irradiation. (**b**) In vitro cell viability for GSNO, APFB, and GAPFB in Hep55.1c with/without NIR laser irradiation. (**c**) Flow cytometry expression of PI and Annexin V for apoptosis. (**d**) Quantitative results of control, APFB, and GAPFB. (**e**) Kyn inhibition ratio of 1-M-DT, GSNO-AuNCs-1-M-DT, and GSNO-AuNCs-1-M-DT with NIR laser. Data are expressed as mean ± SD, *n* = 3. Two-tailed Student’s test was used. * *p* < 0.05, ** *p* < 0.01.

In order to investigate the cytotoxicity of GAPFB, Hep55.1c cells were incubated with different concentrations (25, 50, and 100 μg/mL) of AuNCs and GAPFB for 24 h. As shown in Figure 5a, the AuNC group showed negligible cytotoxicity even under a higher concentration of 100 μg/mL. As the cells were incubated with GAPFB, it was found that the cell viability presented a slight decrease when increasing the GAPFB amount, which may be attributed to self-degradation of GSNO because of a higher concentration of GSNO in the GAPFB group with 100 μg/mL. Therefore, 50 μg/mL was used in the following experiments. Furthermore, to explore the PTT/NO combination treatment on the cell viability of Hep55.1c cells, GSNO, APFB, and GAPFB in the absence/presence of NIR laser were conducted. Figure 5b showed the cell viability of Hep55.1c cells treated in GSNO, APFB, and GAPFB without NIR light irradiation as 85.4 ± 3.4%, 84.3 ± 3.0%, and 83.5 ± 4.4%, respectively. The result illustrated that the groups showed no obvious differences in cell viability and maintained the cell survival rates around 80%. However, the cell viability in the GSNO-treated group decreased to 70.9 ± 4.3% due to the slight self-degradation of GSNO. However, the cell viability in the APFB group decreased to 53.4 ± 2.0% after NIR laser treatments, illustrating the contribution of a single PTT therapeutic effect for killing the tumor cell. Moreover, as the cells were subjected to the combination treatment of PTT/NO with the GAPGB group, the cell viability significantly decreased to 33.1 ± 5.3%, indicating that the PTT/NO combination displayed a significant cytotoxic effect compared to either NO gas or PTT alone. Furthermore, we confirm the effect of NO gas on cell apoptosis in Figure 5c,d. The results showed that the control, APFB and GAPFB caused 16.0 ± 3.8%, 36.2 ± 2.4%, and 47.9 ± 4.0% of the apoptosis, respectively. As shown in Figure 5c, the early stage of apoptosis was mainly induced from the photothermal effect of AuNCs in APFB, which increased about 20% compared to the control group. The late stage of apoptosis was mainly attributed to the reactive nitrogen species (RNS) produced by high-concentration NO generated from the GAPFB, causing destructive activity to the tumor cell. The results indicated that the PTT/NO displayed a synergistic effect for killing tumor cells. Moreover, to confirm the synergistic effect of IDO inhibitor with NO gas, we observed the kynurenine (Kyn) release from the Hep55.1c cells. Since the IDO can produce Kyn from the degradation of tryptophan (Trp), the concentration of Kyn could be decreased under the inhibition of IDO. The Kyn ELISA kit was further used to measure the Kyn concentration and calculate the Kyn inhibition rate [29,30] as shown in Figure 5e. The Kyn inhibition rate of the pure 1-methyl-D-tryptophan (1-M-DT), APFBD, GAPFBD, and GAPFBD with NIR laser was measured as 15.0 ± 3.6%, 16.5 ± 3.1%, 30.1 ± 4.9%, and 63.6 ± 2.4%, respectively. The APFBD nanoparticle with 1-M-DT showed no obvious difference in Kyn inhibition compared to pure 1-M-DT, indicating that the 1-M-DT conjugated on the nanoparticle can maintain the same biofunction as 1-M-DT alone. However, the APFBD with GSNO loaded (i.e., GAPFBD) can increase the inhibition rate by about 15% compared to the APFBD, which illustrated the potential role of NO donors in decreasing the activity of IDO. More surprisingly, GAPFBD with NIR laser showed a higher inhibition rate due to the synergistic effect of a 1-M-DT release and the produced NO gas after NIR laser heating. The result demonstrated that the NO gas production accompanied with 1-M-DT release under PTT can significantly increase the Kyn inhibition rate, revealing the great potential effect of using the PTT/NO/IDO nanoplatform.

### 2.5. In Vitro DCs Maturation Efficiency of PTT/NO/IDO Synergy

**Figure 6:**
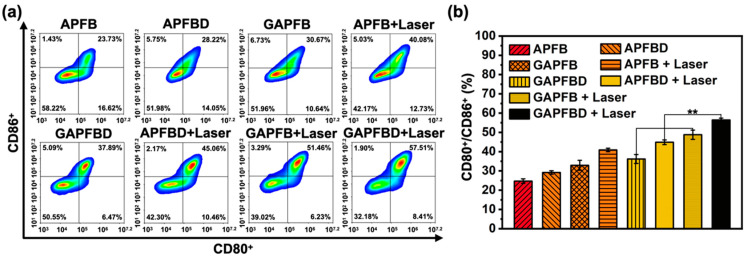
Analysis of immune responses induced by PTT/NO/IDO therapeutic nanoparticle. (**a**) Flow cytometry expression of CD80^+^ and CD86^+^ on mature DCs surface. (**b**) Quantitative results. Data are expressed as mean ± SD, *n* = 3. Two-tailed Student’s test was used. ** *p* < 0.01.

Dendritic cells (DCs), the most potent antigen-presenting cells of the immune system, can activate naive T cells and induce immune memory responses in cancer [31]. Normally, the expression of CD80^+^ and CD86^+^ was used to evaluate the maturation of DCs [32]. To analyze the efficient antitumor immune response induced by PTT/NO/IDO nanoplatform, we investigated the maturation of DCs treated under different conditions of (PTT (APFB + NIR laser), NO (GAPFB), IDO (APFBD), PTT/NO (GAPFB + NIR laser), PTT/IDO (APFBD + NIR laser), NO/IDO (GAPFBD), and PTT/NO/IDO (GAPFBD + NIR laser)) using the flow cytometry in Figure 6. The expression of CD80^+^/CD86^+^ of control (DCs) and DCs + tumor with/without NIR laser was measured as shown in Figure S5. As the Hep55.1c was treated with APFB, the expression of CD80^+^/CD86^+^ was nearly equal to the control group because the APFB contained no drug or without treatments. After being treated with APFBD and GAPFB, it was found to be only 5% and 8% higher than APFB, respectively, indicating that the nanoparticle with a single IDO inhibitor or GSNO displayed poor DC maturation. However, the Hep55.1c treated with APFB and NIR laser irradiation presented 1.6-fold higher expression of CD80^+^/CD86^+^ than APFB, illustrating that the nanoparticle with a single PTT played a crucial role in DC maturation. To evaluate the combination of IDO inhibitor and GSNO with PTT, NIR laser was applied to both groups of APFBD and GAPFB. The CD80^+^/CD86^+^ expression was increased by 10% (APFBD + Laser) and 13% (GAPFB + Laser), more than APFB with single PTT, which demonstrated the synergistic effect from the contribution of IDO inhibitor and GSNO. Moreover, the GAPFBD with NIR laser can simultaneously increase the NO production and enhance the release of 1-M-DT, leading to a 20% increase of the CD80^+^/CD86^+^ compared to GAPFBD alone. The result confirmed that GAPFBD with the synergistic effects of PTT/NO/IDO can largely promote the maturation of DCs because PTT/NO can induce the tumor-associated antigens (TAAs) accompanied with the inhibition of IDO by 1-M-DT.

## 3. Materials and Methods

### 3.1. Materials

Ethylene glycol, silver nitrate (AgNO_3_), PVP (powder, average Mw~29000), Na_2_S·9H_2_O, HAuCl_4_·3H_2_O, sodium chloride, polyacrylic acid (PAA) (Mw = 1800), S-nitrosoglutathione (GSNO, 97%), iron(II) iron(III) oxide (Fe_3_O_4_), Betanin, 1-methyl-D-tryptophan (1-M-DT, 95%), N-(3-dimethylaminopropyl)-N′-ethylcarbodiimide hydrochloride (EDC, 98%), N-hydroxysuccinimide (NHS, 98%), nitric oxide multispecies ELISA Kit (Thermo Fisher Scientific, Waltham, MA, USA), Kynurenine (Kyn) ELISA Kit (FineTest, Wuhan, China), phosphate buffered saline (PBS), CellTiter 96^®^ AQueous One Solution Cell Proliferation Assay (MTS reagent) (Promega Co., Madison, WI, USA), Dead Cell Apoptosis Kit with Annexin V Alexa Fluor™ 488 & propidium iodide (PI) (Invitrogen, St. Louis, MO, USA), Alexa Fluor^TM^ Plus 750 Phalloidin (Invitrogen, Waltham, MA, USA).

### 3.2. Preparation of Gold Nanocages (AuNCs)

The procedure of synthesizing AuNCs followed Xia’s group publication reported in 2007 [33]. First, the silver nanocage (AgNC) solution and the PVP solution (9 mM, 10 mL) were added into the double-necked flask, then HAuCl_4_ solution (0.2 mM) was titrated to the mixture at a rate of 10–15 mL/h. To obtain a desirable LSPR peak (808 nm) of AuNCs, a UV-visible spectrum (Evolution 300, Thermo Fisher Scientific) was used to ensure the stop point of the reaction. Next, sodium chloride (NaCl) was added into the mixture until supersaturation, and the supernatant was collected and centrifuged three times at 11,000 rpm for 10 min. AuNCs were collected in DI water and stored at 4 °C.

### 3.3. Synthesis of GSNO-AuNCs-PAA (GAP)

Synthesis of GSNO-AuNCs-PAA can be divided into two steps. First, the procedure of coating AuNCs with PAA followed Feng’s group publication reported in 2019 [34]. The PAA (25% (*v*/*v*, 10 μL) was added to 10 mL of AuNCs in NaOH solution (0.5 M, 40 μL), then shaken (100 rpm) at room temperature for 3 h. After the reaction was stopped, the solution was centrifuged three times at 10,000 rpm for 10 min. AuNCs-PAA were collected in pH 6.5 phosphate buffer (5 mL) and stored at 4 °C. Second, 1 mL of GSNO (1 mg/mL) was mixed with 5 mL of AuNCs-PAA suspension (5 mg/mL in pH = 6.5 phosphate buffer) and shaken (100 rpm) at 4 °C in the dark overnight. After the reaction stopped, the solution was centrifuged three times at 10,000 rpm for 10 min. GSNO-AuNCs-PAA was re-dissolved in a phosphate buffer (pH 7.4, 1 mL) and stored at 4 °C.

### 3.4. Synthesis of GSNO-AuNCs-PAA-Fe_3_O_4_-Betanin-1-M-DT (GAPFBD)

Synthesis of GSNO-AuNCs-PAA-Fe_3_O_4_-betanin-1-M-DT (GAPFBD) was divided into two steps, which were the synthesis of GAPFB and grafted 1-methyl-D-tryptophan (1-M-DT) onto the surface of GAPFB. First, 2 mL of iron oxide (1 mg/mL) and 1 mL of GSNO-AuNCs-PAA suspension (1 mg/mL) were mixed and shaken overnight under 4 °C in the dark. Then, the ungrafted iron oxide was removed using a micro-separation magnetic holder and GAPF was collected. Second, 2 mL of betanin (10 mg/mL) and 1 mL of GAPF suspension were mixed and shaken overnight under 4 °C in the dark. The ungrafted betanin was removed using a micro-separation magnetic holder. GAPFB was re-dissolved in pH 6.0 phosphate buffer and stored under 4 °C. Next, The EDC (1.6 mM) was added to the GAPFB solution and shaken in the dark for 1 h, then NHS (4 mM) and 1-M-DT were added (1 mg/mL) into the mixture and shaken for 4 h in the dark. Next, the ungrafted 1-M-DT was removed using the micro-separation magnetic holder. GAPFBD was re-dissolved in a pH 6.0 phosphate buffer and stored under 4 °C.

### 3.5. Characterization of GAPFBD

The morphology of AuNCs and GAPFBD were observed using a scanning electron microscope (SEM) and transmission electron microscope (TEM). The zeta potential and particle size of AuNCs, GAP, GAPF, GAPFB, and GAPFBD were measured by electrophoretic light scattering and dynamic light scattering (ELS and DLS; Delsa Nano C particle analyzer, Beckman Coulter, Brea, CA, USA).

### 3.6. Photothermic Effect and Stability of GAPFBD

The GAPFBD solution (AuNCs, 50 μg/mL) was irradiated under 808-nm laser (LSP-PS-FA, Lasever, Ningbo, China) (0.5 W/cm^2^), and the heating curve plot was recorded by the temperature value of every 30 seconds for a period of 15 min. The GAPFBD solution (AuNCs, 50 μg/mL) was irradiated by 808-nm laser (0.5 W/cm^2^) for 15 min, then, the laser turned off to cool down to room temperature. This cycle was repeated three times.

### 3.7. Drug Loading Ability

The suspension of GAP was collected to measure the absorbance at 335 nm by ELISA reader (DV990BV4, GDV, Milan, Italy). The S-nitroso group in the AuNCs was retrospectively calculated using the standard curve [15], and the encapsulation ratio (ER%) was estimated by the following formula [35].

ER% = [(the original amount of sample solution-amount of supernatant after centrifugation)/original amount of sample solution] ×100%.

### 3.8. NO Production of GAPFB

GSNO powder was dissolved in deionized water (50 μg/mL) and placed at 4 °C for three days before the experiment of NO production. GSNO and GAPFB (50 μg/mL) were irradiated by the 808-nm laser (LSP-PS-FA, Lasever, Ningbo, China) (0.5 W/cm^2^, 10 min), and subsequently, the supernatants were collected for NO analysis. During the experiment, the laser with a square spot size of 4 mm^2^ was used to irradiate the 1 mL of the solution containing the collected supernatants. After the irradiation at 0.5 W/cm^2^ for 10 min, the irradiated fluid and the biological solution without laser were measured by the Nitric Oxide Assay Kit. The absorbance values at 540 nm were used to estimate the release amount based on the standard curve (Figure S4).

### 3.9. GSNO Release of GAPFB

GAPFB (50 μg/mL) was placed in three pH values of phosphate buffer solutions (7.4, 6.5, 5.0, respectively) and shaken at 37 °C. The supernatant was collected at 1, 2, 4, 8, 12, 24, 48, and 72 h. The concentration of GSNO was measured by Microplate Spectrophotometer (BioTek, Agilent, Santa Clara, CA, USA) at the absorbance of 335 nm.

### 3.10. 1-M-DT Release of GAPFBD

GAPFBD (50 μg/mL) was placed in three pH values of phosphate buffer solutions (7.4, 6.5, and 5.0, respectively) and shaken at 37 °C. GAPFBD under pH value of 6.5 and 7.4 were irradiated by the 808-nm laser (LSP-PS-FA, Lasever, Ningbo, China) (0.5 W/cm^2^, 10 min) after 24 h. The supernatant was collected at 1, 2, 4, 8, 12, 24, 48, and 72 h. The concentration of 1-M-DT was measured by microplate spectrophotometer (BioTek, Agilent, Santa Clara, CA, USA) at the absorbance of 223 nm.

### 3.11. Cell Culture

Hep55.1c (RRID: CVCL_5766) mouse liver cancer cell line was provided by China Medical University in Taiwan. Hep55.1c was cultured with DMEM medium supplemented with 10% fetal bovine serum (FBS) and 1% penicillin-streptomycin in a 37 °C, 5% CO_2_ incubator. Trypsin was applied on Hep55.1c for subculture. Bone marrow cells which were extracted from thigh bones of 6–8-week-old C57BL/6 WT mice were induced to differentiate to dendritic cells (DCs) in 5 to 6 days culture in RPMI 1640 medium supplemented with 10% FBS, 1% sodium pyruvate, 1% nonessential amino acids, 1% penicillin/streptomycin, GM-CSF (20 ng/mL), and IL-4 (10 ng/mL). The cells were cultured in a complete medium at 37 °C in a humidified atmosphere with 5% carbon dioxide in the air.

### 3.12. Cytotoxicity Assay of Hep55.1c Treated with Nanoparticle

The cell viability was tested by MTS Assay Kit. The cell suspension was added to a 96-well plate with 10^4^ cells/well and placed in an incubator (37 °C, 5% CO_2_) for 24 h. Next, the different concentrations of the AuNCs and GAPFB (AuNCs, 25, 50, and 100 μg/mL) were added and incubated for 24 h. A medium containing 10% MTS reagent was added to each well and incubated for 2 h, then the optical absorbance was quantified at 490 nm using a microplate spectrophotometer (BioTek, Agilent, Santa Clara, CA, USA).

### 3.13. Cell Uptake

The cells were suspended at 10^5^ cells/well into a 24-well plate and placed in an incubator (37 °C, 5% CO_2_) for 24 h. The GAPFBD (50 μg/mL) was added and incubated for 1, 4, 12, and 24 h. The cell suspension was centrifuged (1500 rpm, 5 min) three times for washing, then cell uptake was observed by flow cytometry. For fluorescent microscope, the cell suspension with 10^5^ cells/well and coverslip were put into a 24-well plate, then placed in an incubator for 24 h. The nanoparticle was mixed with culture medium and added into each well for 1, 4, 12, and 24 h. The cells were fixed with 3.7% paraformaldehyde in PBS for 30 min and reacted with 0.25% Triton x-100 in PBS for 15 min. Next, the cells were blocked with 2% BSA in PBS for 1 h, and then the cytoskeleton was stained with Alexa Fluor^®^ 750 Phalloidin (5 unit/mL) for 1 h. Finally, the cells were mounted on a clean glass slide by DAPI-containing mounting medium (Sigma, Saint Louis, MO, USA). The fluorescence images were observed by confocal microscopy (D-eclipse C1; Eclipse, TE2000-U, Nikon, Tokyo, Japan).

### 3.14. NO/Photothermal Treatment of Hep55.1c Cell

The cell suspension was added with 10^5^ cells/well to a 24-well plate and placed in an incubator for 24 h. Next, the nanoparticles of each group (GSNO, APFB, and GAPFB) were added to each well and incubated for 24 h. Hep55.1c cells were then treated with an 808-nm laser (LSP-PS-FA, Lasever, Ningbo, China) (0.5 W/cm^2^, 10 min) or no treatment as a control. After treatment, the cells were cultured for another 24 h. The medium containing MTS reagent (10%) was added to each well and incubated for 2 h, then the optical absorbance was quantified at 490 nm using a microplate spectrophotometer (BioTek, Agilent, Santa Clara, CA, USA).

### 3.15. Apoptosis Analysis of GAPFB

Hep55.1c cell suspension with a concentration of 10^5^ cells/well was added to a 24-well plate and placed in the incubator for 24 h. Next, the nanoparticles of each group (APFB and GAPFB) were added to each well and incubated for 24 h. Hep55.1c cells were then treated with an 808-nm laser (LSP-PS-FA, Lasever, Ningbo, China) (0.5 W/cm^2^, 10 min). After treatment, the cells were cultured for another 24 h. The cells were removed by 0.25% trypsin with 0.02% EDTA. The cell suspension was centrifuged at 1500 rpm for 5 min and re-dissolved in 1× annexin-binding buffer. Next, Alexa Fluor 488 annexin V and PI working solution cell apoptosis reagents were added to react for 15 min at room temperature. Finally, 1× annexin-binding buffer was added to the mix, and apoptosis analysis was performed using flow cytometry (Novocyte^®^, ACEA biosciences, San Diego, CA, USA).

### 3.16. Kyn Inhibition 

A cell suspension with 10^5^ cells/well was added to a 24-well plate, placed in a box, and incubated at 37 °C and 5% CO_2_ for 24 h to stabilize the cell growth. Next, the nanoparticles of each group (1-M-DT, APFBD, and GAPFBD) were added to each well and incubated at 37 °C and 5% CO_2_ for 48 h [36]. After that, the Hep55.1c cells were treated with the 808-nm laser (LSP-PS-FA, Lasever, Ningbo, China) (0.5 W/cm^2^, 10 min) or without any treatment. After the treatment, the cells were cultured for another 24 h. The supernatant was collected as sample reagents after centrifuging cell culture supernatant for 20 min at 1000× *g* and running the assay procedure by Kyn ELISA Kit. The O.D. absorbance was read at 450 nm in the microplate spectrophotometer (BioTek, Agilent, Santa Clara, CA, USA) to measure the inhibition rate.

### 3.17. Analysis of Mature DCs Surface-Marker Expression

The transwell system was used for co-culturing Hep55.1c cells and dendritic cells (DCs), and the DCs were cultured according to the study of Alka et al. [37]. On the fifth day, Hep55.1c cell suspension with 10^5^ cells/well was added to the upper chamber of the transwell and placed in the incubator for 24 h. Next, the nanoparticles of each group (APFB, GAPFB, and GAPFBD) were added to the upper chamber of the transwell and incubated for 24 h. Hep55.1c cells were then treated with an 808-nm laser (LSP-PS-FA, Lasever, Ningbo, China) (0.5 W/cm^2^, 10 min), and the cells were cultured for another 4 h. After culturing the cells extracted from the mouse thigh bone for six days, the non-adherent primary bone marrow dendritic cells (BMDC) were collected by gently pipetting the culture medium. The BMDC cell suspension with 2 × 10^5^ cells/well was added to the lower chamber of the transwell and placed in the incubator for 20 h. To analyze the expression of cell surface markers, BMDC was collected to centrifuge and wash with PBS at 250× *g* for 8 min three times. The BMDC were stained with anti-CD11c-FITC (RUO), anti-CD80-PE (16-10A1), and anti-CD86-PE (GL1) at 4 °C in the dark for 30 min. The DC phenotype was evaluated by flow cytometry, and the result was expressed by the distribution of fluorescence intensity.

## 4. Conclusions

In this study, we have successfully developed a dual-sensitive GAPFBD nanoparticle as a single nano-platform to achieve combination therapy of photothermal therapy (PTT), NO gas therapy, and IDO immunotherapy. The results proved this dual-sensitive nanoparticle can co-deliver and precisely release GSNO and 1-M-DT through the control by 808-nm laser under a certain pH environment, leading to tumor destruction. The synergistic effect has demonstrated the efficiently anti-tumor therapeutic effects and the great potential of producing the tumor-associated antigens (TAAs) using the GAPFBD to stimulate the maturation of dendritic cells (DCs), leading to long-term immunity. Further, the excellent achievement clarified in this study makes it a promising choice for in vivo studies and clinical applications in combination cancer therapies.

## Data Availability

The data presented in this study are available on request from the corresponding author.

## Supplementary Materials

The following are available online at https://www.mdpi.com/article/10.3390/ph15020138/s1, Figure S1: Elemental analysis. (a) GAP and (b) GAPFBD, Figure S2: Standard curve of (a) GSNO and (b) 1-M-DT, Figure S3: (a) GSNO and (b) 1-M-DT stability of GAPFBD under storge condition of pH = 7.4 and pH = 6.0, Figure S4: Standard curve of nitrite concentration, Figure S5: Analysis of immune responses induced by PTT/NO/IDO therapeutic nanoparticle. (a) Flow cytometry expression of CD80+ and CD86+ on mature DCs surface. (b) Quantitative results, Table S1: Element analysis of the GAPFBD.
