# Supplementary material for: Dual-Sensitive Gold-Nanocubes Platform with Synergistic Immunotherapy for Inducing Immune Cycle Using NIR-Mediated PTT/NO/IDO
Source: Pharmaceuticals (Basel). 2022 Jan 25;15(2):138. doi: 10.3390/ph15020138 (PMC8880079; doi:10.3390/ph15020138)
Supplement: Supplementary file 1 [file pharmaceuticals-15-00138-s001.zip › pharmaceuticals-1553913-supplementary.pdf]

Supplementary Information

Dual-sensitive Gold-nanocubes platform with synergistic immunotherapy for inducing immune cycle using NIR-mediated PTT/NO/IDO

Hsin-Yi Tsao[1,†], Hung-Wei Cheng[1,†], Chia-Chi Kuo[1], and San-Yuan Chen [1,2,3,4,*]

[1] Materials Science and Engineering, National Yang Ming Chiao Tung University, 300 Hsinchu, Taiwan

[2] Graduate Institute of Biomedical Science, China Medical University, 404 Taichung, Taiwan

[3] Frontier Research Centre on Fundamental and Applied Sciences of Matters National Tsing Hua University, 300 Hsinchu, Taiwan

[4] School of Dentistry, College of Dental Medicine Kaohsiung Medical University Kaohsiung, 807378, Taiwan

* Correspondence: sanyuanchen@nycu.edu.tw

† These first authors contributed equally to this work.

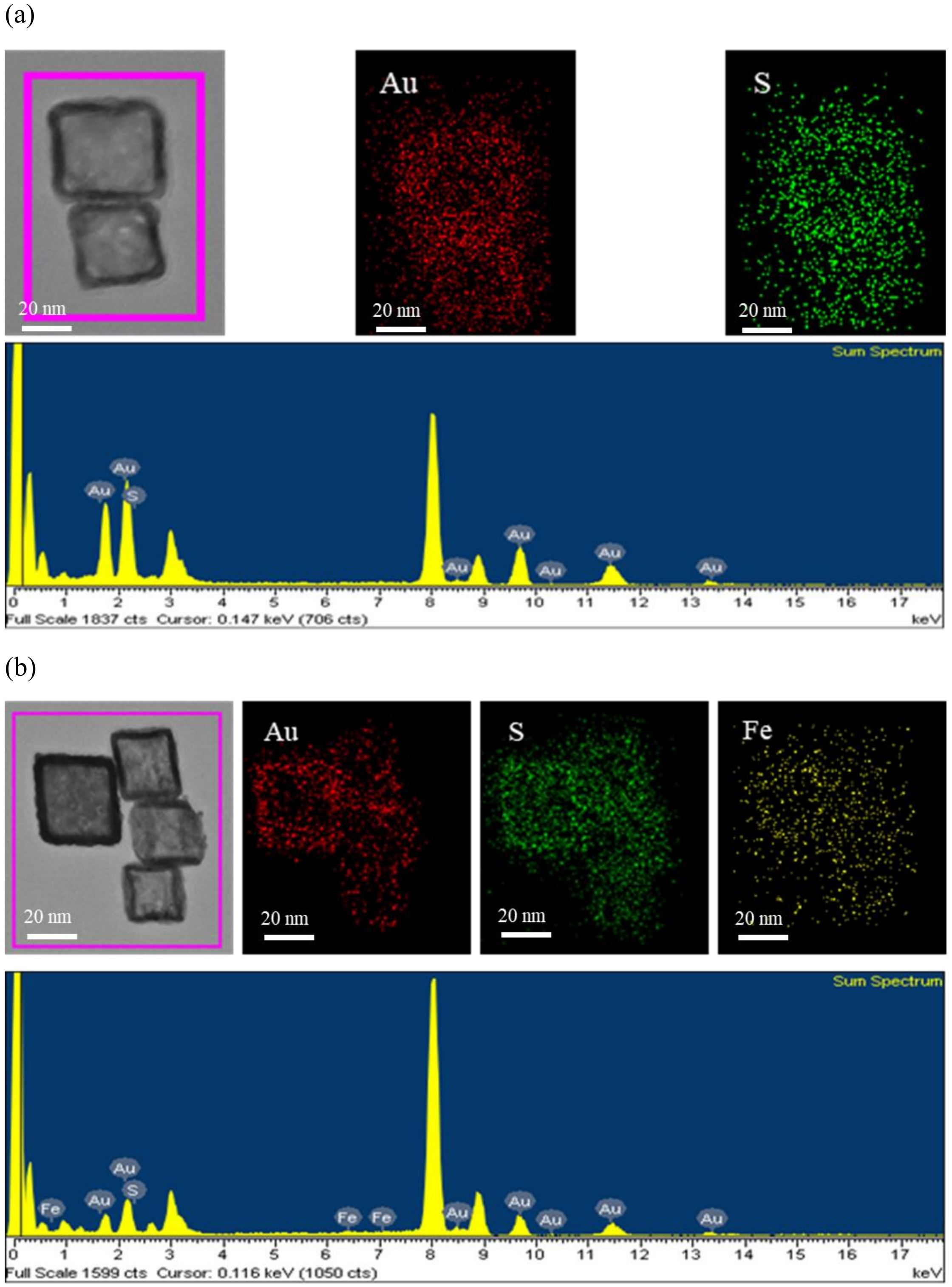

Figure S1. Elemental analysis. (**a**) GAP and (**b**) GAPFBD.

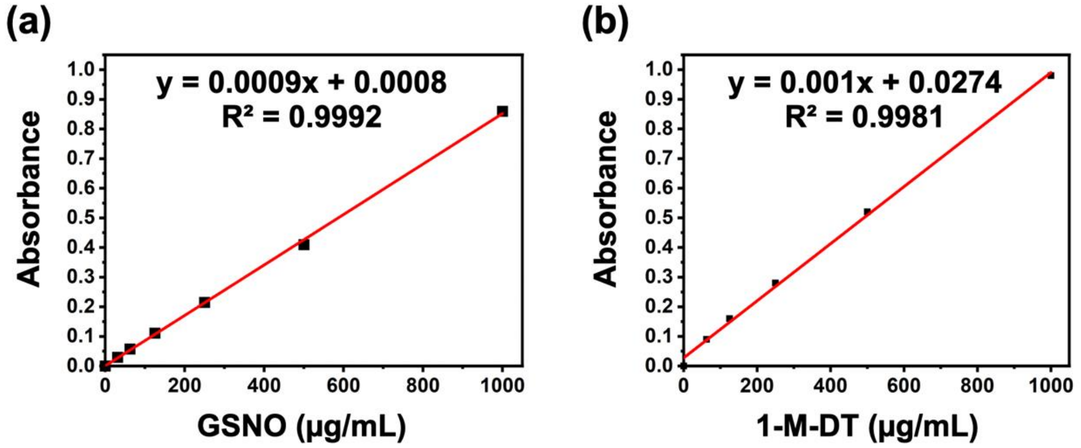

Figure S2. Standard curve of (**a**) GSNO and (**b**) 1-M-DT.

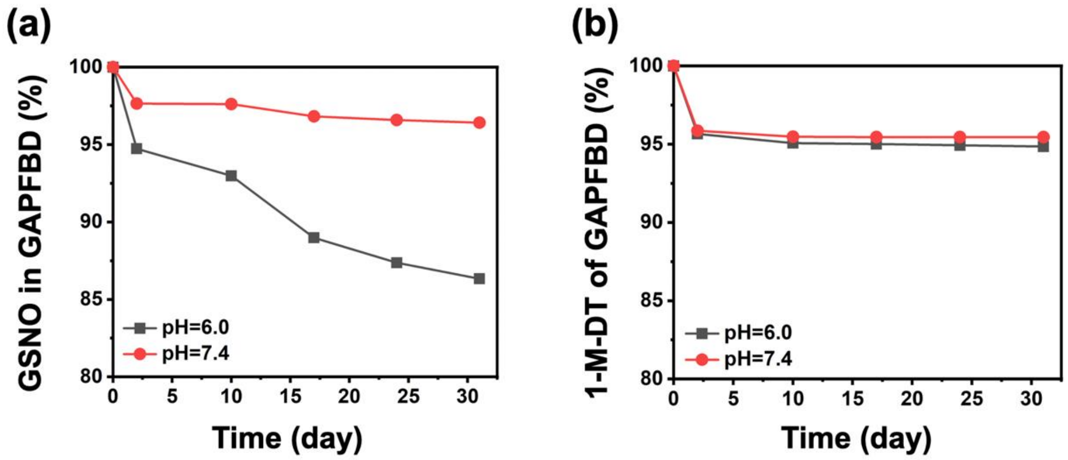

Figure S3. (**a**) GSNO and (**b**) 1-M-DT stability of GAPFBD under storge condition of pH=7.4 and pH=6.0.

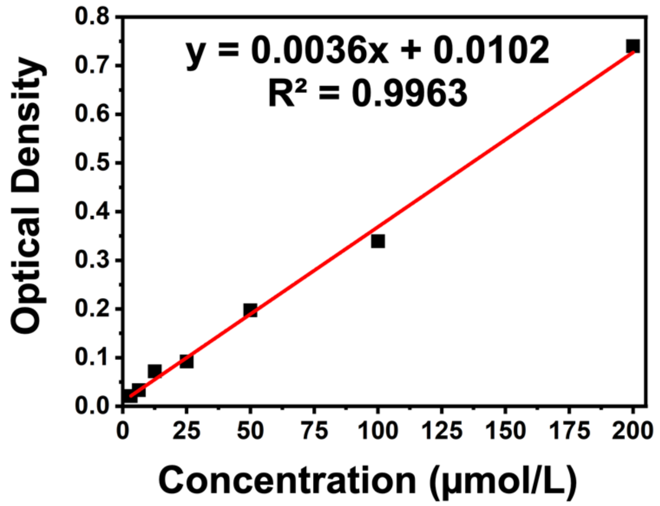

Figure S4. Standard curve of nitrite concentration.

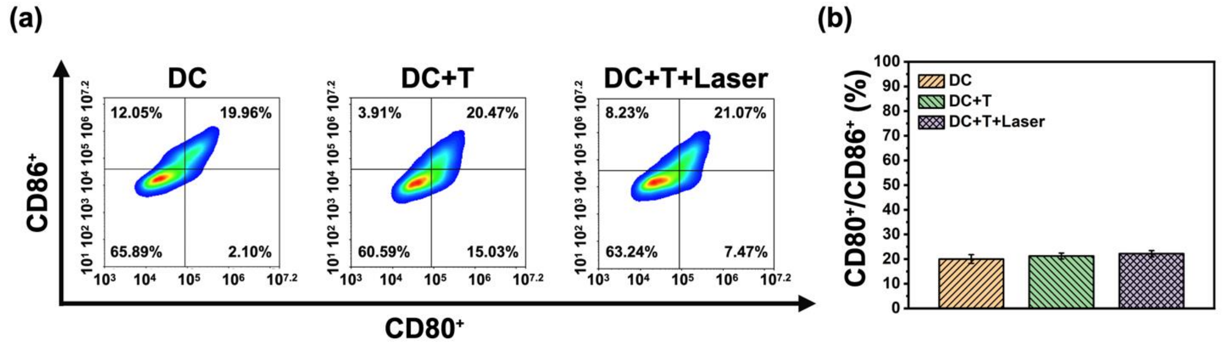

Figure S5. Analysis of immune responses induced by PTT/NO/IDO therapeutic nanoparticle. (**a**) Flow cytometry expression of $CD80^+$ and $CD86^+$ on mature DCs surface. (**b**) Quantitative results. Data are expressed as mean ± SD, n=3.

Table S1. Element analysis of the GAPFBD.

**Element**	**Atomic%**
S	9.14
Fe	5.48
Au	85.38
Totals	100
